# Childhood Lead Exposure Associated with the Use of Kajal, an Eye Cosmetic from Afghanistan — Albuquerque, New Mexico, 2013

**Published:** 2013-11-22

**Authors:** Leilani Schwarcz, Crystal L. Begay, Lance A. Chilton, J. Brian Shirley, Steven A. Seifert

**Affiliations:** Environmental Health Epidemiology Bur, New Mexico Dept of Health; Dept of Pediatrics; New Mexico Poison and Drug Information Center, Univ of New Mexico

Lead is a toxic metal that damages blood cells, the kidneys, the cardiovascular system, and the developing nervous system. The risk for lead exposure causing subsequent cognitive and neurobehavioral deficits is especially high among toddlers because of their hand-to-mouth activities and their higher absorption of ingested lead compared with adults (1). In January 2013, the New Mexico Department of Health (NMDOH) received a report from an Albuquerque clinic of a refugee child aged 20 months (patient 1) with an elevated blood lead level (BLL) of 27.0 *μ*g/dL (CDC reference value = 5.0 *μ*g/dL). Medical staff informed NMDOH that the child and family used kajal, a traditional eye cosmetic brought from Afghanistan, their country of origin. Further investigation revealed that patient 1’s brother, aged 4 months (patient 2), also had an elevated BLL of 33.5 *μ*g/dL. Laboratory analysis of kajal used by the family showed a lead content of 54%. These two cases highlight the potential for lead poisoning among refugee populations in the United States and call attention to contaminated consumer products as a source of lead exposure. Physicians who provide health services to refugee and immigrant children should be aware of this potential exposure. Health-care providers who routinely screen refugee and immigrant children for elevated BLLs should consider asking questions about the use of traditional eye cosmetics.

In January 2013, in preparation for a preschool program, patient 1 was screened for lead and had a capillary blood lead test result of 27.0 *μ*g/dL. Two weeks later, confirmatory venous blood lead testing of patients 1 and 2 showed BLLs of 18.9 *μ*g/dL and 33.5 *μ*g/dL, respectively; both results exceeded CDC’s current reference value of 5.0 *μ*g/dL.* The children’s cousin, aged 3 years, also was tested and found to have a venous BLL of 5.3 *μ*g/dL. All three children were asymptomatic, as reported by their physicians. Communication with the family indicated that the cultural practice of applying kajal to the children’s eyelids was intended to promote eye health. Other traditional eye cosmetics (i.e., surma, tiro, and kohl) are widely used in Asia, Africa, and the Middle East and have been implicated as sources of lead poisoning (2–4). The children’s physician recommended that the parents discontinue use of the eye cosmetic, and if continued use of eyeliner was desired, that they replace it with an over-the-counter cosmetic obtained in the United States.

On receipt of the elevated blood lead test results, NMDOH interviewed the family to investigate potential sources of lead exposure. Based on information about the family’s residential conditions, the parent’s occupations, and family hobbies, the use of kajal was suspected as the main source of the lead exposure. The use of an imported curry powder (gutti) was suspected as a secondary source. Because of the mother’s simultaneous use of kajal during breastfeeding, the children’s physician recommended a blood lead test for her. Venous lead testing of the children’s mother showed a BLL of 6 *μ*g/dL.

The kajal and the curry powder were collected and analyzed for lead content using Environmental Protection Agency method 200.8.† Quantitative analysis of the kajal found 54% lead by weight, and the curry powder contained 0.01% lead by weight. At the time of initial lead screening, the family had lived in an apartment in Albuquerque for approximately 2 months. Based on responses obtained from the questionnaire, no housing-related lead exposure sources could be identified. NMDOH ruled out other potential lead hazards from occupational exposures, kitchen utensils, hobbies, toys, inexpensive jewelry, pica, or neighborhood sources. The kajal and curry powder were brought with the family from Afghanistan. The primary lead exposure was likely a combination of dermal and conjunctival absorption and ingestion of lead-containing kajal from hand-to-mouth transfer (2).

Patients 1 and 2 were found to be healthy, with no apparent developmental delays or medical problems. In accordance with CDC guidelines, follow-up testing was performed on both patients approximately 1 month after the initial confirmatory testing. Patient 1’s venous BLL dropped to 17.7 *μ*g/dL, from 18.9 *μ*g/dL. Patient 2’s venous BLL dropped to 28.2 *μ*g/dL, from 33.5 *μ*g/dL. Four months after discontinuing use of kajal, patient 2’s venous BLL further declined to 22.1 *μ*g/dL and patient 1’s venous BLL declined to 14.7 *μ*g/dL. Monitoring of the children’s BLLs will continue until their BLLs are <10.0 *μ*g/dL for 3 months, in accordance with current state guidelines.§

## Editorial Note

These cases highlight the risk for pediatric lead exposure among refugee populations and draw attention to the potential exposures to lead-contaminated imported products. Historically, most U.S. childhood lead poisoning cases have been associated with ingestion of chips and dust from lead-based paint sources (5,6). Lead poisoning from nonpaint sources, including folk remedies (5,7), imported goods (8), toys (9), and food (8), also have been reported in recent years. Global free trade, immigration, and the frequency of international travel have contributed to the potential exposure of children in the United States to nonpaint sources of lead in unregulated consumer products, folk remedies, herbal supplements, and other cultural paraphernalia (8). As new sources of childhood lead exposure are identified, it is important to document information about these products and how they are used in cultural practices. This information can then be used to develop strategies that address cultural differences and language barriers to minimize health risks associated with lead-contaminated products.

Kajal and similar traditional eye cosmetic preparations have been found to contain lead concentrations as high as 70% and have been documented as sources of childhood lead poisoning for >30 years (2,3). The cultural significance and availability of these products among refugee and immigrant populations and the potential for unintentional toxic exposure pose a substantial public health risk. Despite the FDA import ban on kohl, surma, and kajal, these products still appear in households, transported in personal luggage and distributed illegally by retailers. The risk for high BLLs caused by repeated exposure to multiple lead-contaminated consumer products and accumulation is a concern.

Refugee children have been found to have a higher average BLL at their time of arrival in the United States, as compared with average BLLs measured in U.S. children (10). Education directed to health-care professionals and to refugee and immigrant populations is the prime strategy to prevent lead poisoning. Health-care professionals need to discuss lead exposure prevention with refugee patients and their families. Conveying the health risks from lead exposure in a culturally sensitive manner to patients who also might be stressed by recent immigration can be challenging, especially because not all symptoms of lead toxicity are outwardly apparent. Current CDC recommendations advise that all refugee children aged 6 months–16 years be screened for lead within 90 days of their arrival into the United States, and again 3–6 months after resettlement, regardless of initial testing results (10). Expanding lead screening to all refugee infants might be warranted. Both patients 1 and 2 are refugees; patient 2 was aged 4 months and might have been overlooked had he not had an older sibling who was identified with an elevated BLL.

What is already known on this topic?

Lead poisoning continues to be an important, preventable health problem. The common source of lead exposure in the United States is deteriorating lead-based paint and dust; however, some traditional remedies and cosmetics also contain lead.

What is added by this report?

Two male children in New Mexico, aged 20 and 4 months, were found to have elevated blood lead levels of 27.0 and 33.5 *μ*g/dL, respectively. Investigation implicated kajal, a cosmetic imported from Afghanistan, that was applied as a folk remedy to the children’s eyelids. The kajal was found to contain 54% lead.

What are the implications for public health practice?

Health-care providers who provide health services to refugee and immigrant children, even in small communities, should be aware of the unique lead exposure risk factors among this population. Expanding lead screening to all infants and children, and pregnant women, might avoid unintentionally excluding cases. Clinicians and other health-care workers might reduce the risk for lead exposures by discussing lead exposure hazards with their patients, especially with women receiving prenatal care or during early childhood screening programs.

Pediatricians and other health-care professionals should incorporate lead screening as part of routine medical evaluation of refugee and immigrant children, specifically, those who are relocating from countries with a documented history of increased lead exposure risks associated with cultural practices or unregulated industrial processes. Health communication addressing potential lead hazards should be provided in a culturally sensitive manner. Clinicians and other health-care workers providing services to refugees and immigrants from Africa, Asia, and the Middle East should be aware of potential sources of lead in these populations and ask about the use of traditional eye cosmetics, especially with women receiving prenatal care or during early childhood screening programs, and questions about the use of folk remedies should be included in lead-exposure risk assessment questionnaires. Additionally, public health education campaigns concerning lead exposure risks and geared to refugee and immigrant populations might increase awareness of lead content in traditional eye cosmetics.

## Footnotes

* Additional information available at http://www.cdc.gov/nceh/lead.

† Available at http://water.epa.gov/scitech/methods/cwa/bioindicators/upload/2007_07_10_methods_method_200_8.pdf.

§ Available at http://nmhealth.org/eheb/documents/Lead/Appendix_3_case_mngmnt_guidelines.pdf.
